# Controversies and considerations regarding the termination of pregnancy for Foetal Anomalies in Islam

**DOI:** 10.1186/1472-6939-15-10

**Published:** 2014-02-05

**Authors:** Abdulrahman Al-Matary, Jaffar Ali

**Affiliations:** 1 Department of Neonatology, King Fahad Medical City Riyadh, P.O. Box 59046, 11525 Riyadh, Kingdom of Saudi Arabia; 2 Department of Obstetrics and Gynaecology, Faculty of Medicine, University of Malaya, 59100 Kuala Lumpur, Malaysia

**Keywords:** Abortion, Ensoulment, Islam, Glorious Qur’an, Termination of pregnancy, TOP

## Abstract

**Background:**

Approximately one-fourth of all the inhabitants on earth are Muslims. Due to unprecedented migration, physicians are often confronted with cultures other than their own that adhere to different pdigms.

**Discussion:**

In Islam, and most religions, abortion is forbidden. Islam is considerably liberal concerning abortion, which is dependent on (i) the threat of harm to mothers, (ii) the status of the pregnancy before or after ensoulment (on the 120^th^ day of gestation), and (iii) the presence of foetal anomalies that are incompatible with life. Considerable variation in religious edicts exists, but most Islamic scholars agree that the termination of a pregnancy for foetal anomalies is allowed before ensoulment, after which abortion becomes totally forbidden, even in the presence of foetal abnormalities; the exception being a risk to the mother’s life or confirmed intrauterine death.

**Summary:**

The authors urge Muslim law makers to also consider abortion post ensoulment if it is certain that the malformed foetus will decease soon after birth or will be severely malformed and physically and mentally incapacitated after birth to avoid substantial hardship that may continue for years for mothers and family members. The authors recommend that an institutional committee governed and monitored by a national committee make decisions pertaining to abortion to ensure that ethics are preserved and mistakes are prevented. Anomalous foetuses must be detected at the earliest possible time to enable an appropriate medical intervention prior to the 120^th^ day.

## Background

Approximately one-fourth of all the inhabitants of the earth are Muslims [1]. The current unprecedented movement of people has re-distributed people of all faiths to almost every corner of the globe. Healthcare providers are oftentimes confronted with ethical issues regarding peoples of different cultures, the resolution of which differs from that of their own [2-4].

The inability of service providers to effectively handle sensitive issues such as the termination of pregnancy (TOP) can result in enormous prolonged suffering for both the parents and the affected children. It is therefore crucial for healthcare workers to be sensitive to the norms of different cultures to be able to deal effectively with specific ethical issues. In particular, it is crucial to be adequately acquainted with the norms of the major faiths of the world, especially when dealing with matters of a sensitive nature such as TOP. Islam allows TOP under certain conditions if the pregnancy has not progressed beyond the 120^th^ day of pregnancy, which is also referred to as the day of ensoulment. This is crucial because if a diagnosis is delayed for any reason, then TOP will become illegal if attempted past the 120^th^ day. Delays in diagnosis have contributed to considerable suffering for both parents and the affected children. Both speed and a timely intervention are critical in the management of foetal anomalies in the Muslim patient.

There is now a large volume of compelling empirical evidence [2-29] indicating that considerable pain and suffering is experienced by the mothers of anomalous foetuses because they did not undergo TOP due to cultural reasons or because they were delayed and could not perform a TOP before the 120^th^ day of pregnancy. A review of the literature and anecdotal evidence suggest this predicament to be due to (i) a lack of knowledge on the part of parents, which is compounded by guilty consciousness; (ii) a delay in the early and rapid diagnosis of foetal anomalies due to a flawed or inefficient referral system; and (iii) the lack of expert clerical opinions. Other contributing factors include (iv) the lack of proper guidelines for the healthcare worker to undertake the early diagnosis of foetal anomalies and to adequately handle the TOP before the 120^th^ day of pregnancy appears to contribute to the severity of the problem. All of these may be further compounded if the attending physician is (v) uninformed regarding the cultural norms of the patient. If no intervention is taken before the 120^th^ day of pregnancy the mother will be condemned to carry the anomalous foetus to term, which will be a futile and painful effort.

Policy makers and religious clerics in Islamic countries entrusted with the task of issuing guidelines or edicts on TOP must bear in mind that the suffering felt by mothers of anomalous foetuses is real and extremely stressful. Guidelines that are non-conducive for TOP past 120 days of pregnancy due to a delayed diagnosis have made the task of the healthcare providers very difficult. The termination of a pregnancy due to an anomalous foetus prior to day 120 of a pregnancy constitutes a *period of opportunity* that a women can utilise to safeguard her health and well-being. A missed opportunity could result in prolonged suffering for the affected parents and in particular the mother. The pregnancy cannot be terminated if the *period of opportunity* has been missed, and the anomalous foetus must be carried to term. Anecdotal evidence suggests that a missed opportunity to end a pregnancy for foetal anomaly has the potential to culminate in maternal morbidity or even mortality. It is emphasised that the anguish endured by the affected women is real. This pain and suffering may been avoided if knowledge of the religious edicts on the permissibility of TOP as provided for in the Glorious Qur’an and prevailing Islamic wisdom is readily and widely available and if healthcare workers are well acquainted with such provisions so that they can undertake an appropriate intervention to avert disastrous and futile pregnancies through early diagnosis followed by TOP if appropriate and where permissible.

The ineffectiveness of the current available laws or edicts on TOP is readily evident from the distressing outcomes endured by the Muslim populations of the world due to foetal anomalies [2-29], including even those residing in developed nations [2,4,5,10,16,20,21,23-29].

There are two main sects in Islam, the *Sunni* and the *Shi’ite*. A majority of Muslims belong to the Sunni sect, and the remaining 10 to 15% are Shiites [30]. This article will review the views of both schools of thought. It will attempt to highlight and discuss differences of opinion with regard to TOP between these two sects. Only a few countries apply Islamic principles as the basis of laws (viz: Saudi Arabia and Iran). In these countries, the religious edicts issued by an authorised council are considered law. In the remaining countries, even if Islam is the official religion, the laws are made by a parliament or the royal court, which may or may not conform to religious edicts. The religious edicts issued by an Islamic council of scholars in individual Islamic countries are applied, but such edicts are not law until passed by their parliament or an equivalent authority.

This article will attempt to review the Islamic basis for the permissibility and prohibition of abortion as expounded by Islamic scholars and councils as well as the authors’ opinion. The edicts issued in Saudi Arabia have far-reaching consequences and implications. The Muslim nation looks towards Saudi Arabia for guidance in all matters pertaining to religion. Therefore, this paper will focus on Saudi rulings. This article is intended to assist healthcare providers, decision makers, would-be parents, and health administrators to address or make decisions regarding this very controversial subject. The dreadful outcome of not performing TOP in a timely manner must be addressed and eliminated in the 21st century. The issue of TOP past 120 days of pregnancy must be revisited, debated and resolved to prevent suffering.

### Ethics: The Islamic viewpoint

Islamic law is derived from (i) the Qur’an; (ii) the recorded authentic sayings and precedents set by the Prophet (*Sunnah*) and the prophetic decrees (the compilation or the records of the *Sunnah* are called *Hadith*); and (iii) the *ijtihād*. The ijithad can be defined as the rulings deduced from the Islamic principles based on the Qur’an and *Hadiths* by learned scholars who arrive at religious edicts or *Fatwas* to address a particular situation [3,12].

### When does life begin?

All Abrahamic religions and indeed all religions forbid the taking of a life [31-33]. In Islam, in the context of the embryo, the question arises whether it is a living entity? Logically a lifeless entity feels no pain nor is it sensitive to its environment, and therefore, the ethics of its externally engineered demise is less debatable and therefore less questionable and more permissible than if the entity is a living individual and the need to abort is a necessity. In all Abrahamic religions and in most religions, the question of the soul is pmount [34]. A living individual not only has a physical body but also has a soul. Therefore, it follows that an embryo is not an individual until it has a soul. This, in Islam, is generally believed to take place at approximately 120 days after fertilisation [3,15,34,35] although some schools of thought may argue that life begins the moment the sperm fertilises the egg. On the basis of the *Hadith,* Muslims *a*ssume the former to be correct. It is noteworthy that one school of Islamic thought allows embryo research up to 14 days post fertilisation for the benefit of the advancement of science [19].

An embryo becomes an individual with the full rights of a living person only after it is bestowed a soul, prior to which, it is just an entity, soulless and hence, lifeless as it were, to most Islamic scholars; therefore, it has limited rights but is not devoid of rights. In consideration of these points, the general belief in Islam is that the embryo begins life following ensoulment at day 120 after fertilisation [3,15,34,35]. It is noteworthy that the embryological development of humans is described in detail in the Glorious Qur’an. A detailed description concerning the embryo and foetal development in the Glorious Qur’an appears to have some pllel with current scientific knowledge [36].

### Views of the four *Sunni Madhabs* (Schools of Thought)

*Sunni* Muslims belong to one of the four major schools of thought, which are called *Madhhabs*, viz: *Hanafi; Shafi’i; Maliki;* and *Hanbali*. The *Madhabs* are neither clans nor sects but only schools of thought. These four schools of *Sunni* thought derive their Islamic rules from the Qur’an, the *Hadith* and new rules based on the *Ijtihad.* The generally accepted belief is that abortion is forbidden at any stage of a pregnancy on the basis of the following verses from the Qur’an.

“….. And do not kill the soul which God has forbidden except for the requirements of justice……” [Glorious Qur’an, Al- An’am 8: 151].

Thus, the termination of a pregnancy, even at the earliest possible stage, without medical justification is not allowed (even for social or economic reasons), as stated in the Glorious Qur’an:

“… do not Kill your children for fear of want: We shall provide sustenance for them as well as for you. Verily the killing of them is a great sin.” [Glorious Qur’an, Al- Esraa’ 15: 31].

### Religious edicts and the law

#### The opinions of Sunni scholars on termination of pregnancy (TOP)

The *Hanafi’* and many of the *Shafi’i* schools state that abortion is permissible until the end of four months, only if one has legitimate grounds for abortion (Table 1). While the *Maliki* and *Hanbali* schools state that abortion is permissible at the request of both the parents for up to 40 days with a legitimate cause, this is principally prohibited from day 40 onwards [37,38].

**Table 1:** Views of Muslim Sunni Legal Schools of Thought and Shi’ite Sect on Termination of Pregnancy

**Sects/Schools**	**Sunni Maliki**	**SunniHanbali**	**SunniHanafi**	**SunniShafiie**	**Shi’ite**
Abortion permissible?	No	No	No	No	No
If legitimate when abortion is permissible?	≤40 days	≤40 days	≤120 days	≤120 days	≤120 days

Past and contemporary Islamic scholars opine that abortion is totally forbidden at any stage of a pregnancy. However, some scholars consider it allowed during the stage of *nutfah* (i.e., in the first 40 days) and consider it forbidden thereafter. Others consider TOP allowed in the stages of *nutfah* and *alagah* (i.e., the first 80 days) and consider it forbidden thereafter. Still others consider it allowed during all stages of the first four months provided that there are reasonable grounds for the abortion [39-44].

The Egyptian Grand Sheikh of Al-Azhar, the highest Islamic council in Egypt, issued a religious edict in 1998 and 2004 permitting unmarried women that are victims of rape access to abortion even after 120 days [45,46].

A religious edict from Jordan allowed TOP before 120 days for documented severe foetal anomalies without both parents’ consent. The termination of a pregnancy for a foetal anomaly is also allowed after 120 days if three specialists document these severe anomalies and both parents consent [47].

#### The International Islamic Fiqh (Islamic Jurisprudence) Council

The International Islamic *Fiqh* Council (IIFC; Academy) is an Islamic *Sunni* institution of the World Muslim League that is based in Mecca, Saudi Arabia. Its members are representatives of individual countries where Islam is the predominant religion. They are chosen by their respective governments as the senior-most Islamic scholars of their respective countries. Their main task is to meet on a regular basis to discuss current debatable issues affecting Muslims and to formulate rulings for resolving such issues.

The IIFC has ruled the following:

If proven by a committee of at least two competent and trustworthy medical experts on the basis of medical examinations with the use of appropriate equipment and laboratory findings before 120 days of pregnancy that the foetus has serious anomalies that will be present at birth, only then is it permissible to abort after the request of the parents.

When the pregnancy reaches or is beyond the 120^th^ day, abortion becomes totally forbidden and is deemed a form of murder that will result in prosecution [37,38,48] unless the continuation of the pregnancy to full term poses a risk to the mother’s life; abortion shall then be considered permissible. This decision must be based on the opinions of at least two competent and trustworthy medical experts in the field.

This ruling was based on revelations derived from Glorious Qur’an:

“…No soul shall have imposed upon it a duty but to the extent of its capacity; neither shall a mother be made to suffer harm on account of her child….” [Glorious Qur’an, Al- Baqara 2:233].

#### Current Shi’ite stance on the termination of pregnancy due to foetal anomalies

The Iranian parliament amended its stance [15] as per the recommendations of the Guardian’s Council (that conforms to the principles of the Shi’ite sect) on June 21, 2005 as stated below.

“Therapeutic abortions may be performed under the following conditions. First, the foetus must be less than four months of age, that is, before ensoulment. Second, the foetus must be suffering from profound developmental delay or profound deformations or malformations. Third, these fetal problems must be the cause of extreme suffering or hardship for the mother or the foetus. Fourth, the life of the mother should be in danger. Fifth, both the mother and the father must give their consent to the procedure. The physician performing the abortion shall not be penalized for the performance of these services”.

### Guiding principles in Islam

Islam, in general, has provided broad guiding principles that could be utilised to overcome any dilemma that may arise in life. The first guideline asks one to consider whether a particular action fulfils the basic objectives of jurisprudence. These objectives include the (i) preservation of religion, (ii) preservation of life, (iii) preservation of genealogy or parentage, (iv) preservation of the mind and health, and (v) the preservation of property. The second guideline seeks to prevent a detriment that takes priority over the fulfilment of a benefit or interest [3,15,34,35].

With scientific advancement it is inevitable that healthcare workers will be presented with new and ever progressing knowledge and indeed will be confronted with novel medical and scientific techniques and methods on a continuous basis, all of which can be utilised for the good, to ward off detriment, harm and difficulty. It is incumbent upon every Muslim to seek knowledge and to seek a cure for diseases (because *for every disease there is a cure -* well documented *Hadith).* In the years to come, the technologies at our disposal will provide enormous opportunities to help prevent serious health disabilities. It is crucial that Muslims remain open to new treatment modalities that could help avoid situations that could result in lifelong disability and suffering for the affected individual and that pose a serious difficulty to the immediate family members and a burden on the healthcare system. Indeed Islam intends to neither create difficulties nor expects its followers to endure harm as revealed in the following Glorious Qur’anic verses:

“No soul should be compelled beyond capacity, neither the mother made to suffer for the child nor the father for his offspring…” [Glorious Qur’an, Al-Baqara 2:233]

“God does not burden a soul beyond capacity”. [Glorious Qur’an, Al-Baqara 3:286].

### The current ruling in Saudi Arabia on abortion

The Standing Committee for Scientific Research and for Issuing Edicts, Preaching and Guidance (SC) in Saudi Arabia is a committee of senior Islamic scholars who are allowed to issue edicts. These edicts are considered law in Saudi Arabia if the edict is signed by the majority of the committee members.

In Saudi Arabia, there has been continuous debate on abortion, and the laws have evolved only marginally over the last few decades. In early 2011, the SC of Saudi Arabia issued an edict (Fatwa no. 240 dated 16 January 2011) [49] legalising abortion in certain circumstances. This edict is based on the two Qur’anic verses quoted in the preceding section.

The provisions of the said current edict on abortion in foetal anomalies are appended below:

1. Abortion of a malformed foetus after 120 days of conception or 19 weeks of gestation following ensoulment is permissible if the pregnancy is certain to cause the death of the mother.

2. Abortion of a malformed foetus before 120 days or prior to ensoulment is permissible if it is certain that the foetus will die following birth or if it has severe incurable disabilities.

3. Abortion of the foetus at any stage of pregnancy is permissible if intrauterine death was confirmed.

4. Abortion shall not be permissible under any circumstance without a medical report from a specialised and trustworthy committee composed of at least three competent physicians following written informed consent of both parents or the mother alone if the pregnancy and its continuance was affecting or will affect her health. The consent can be provided by persons delegated by the parents if the parents are not able to provide the same for any valid reason. The signed consent must be retained in the medical records of the mother [49].

## Discussion

Although the Islamic stance on the abortion of malformed foetuses has remained mainly unaltered, the current edict from the SC of Saudi Arabia provides a large margin of comfort to treating physicians as it removes guilt if they were to pursue a course of treatment involving abortion of a malformed foetus prior to ensoulment. Rule number two above states that abortion is permissible prior to ensoulment if the malformed or defective condition of the foetus is incurable. A large number of foetal anomalies are considered incurable and thus may be amenable to therapeutic abortion. More importantly, under this edict the mothers are empowered to decide on their own whether to proceed with TOP, which provides significant freedom to the mother to safeguard her well-being irrespective of external influences. The freedom is further extended to assist mothers and parents in making decisions through the delegation of authority so that if for any reasons the mothers or parents are not able to make the decision the delegated individual(s) can make the decision to proceed with TOP.

Religious edicts, in general, are not law in most Islamic countries except in Saudi Arabia and Iran (Table 2). In all other countries edicts are merely guidelines unless their parliaments rule it be made a law. There is a need to streamline this issue. Edicts must be debated by relevant authorities and made law if justified or abandoned if found unsuitable.

**Table 2:** Religious edicts, Islamic law and practice of TOP in Islamic countries

**Country**	**Az Azhar, Egypt**	**Jordan**	**IIFC**	**Iran (Shi’ite)**	**Saudi Arabia**	**All/other Islamic countries**
**Edict/Law**	Edict*	**Edict**	**Edict**	**Law**	**Law**	**Common law/edicts/Clandestine practices**
**When TOP permissible?**	Rape victims severe foetal anomaly (SFA)	SFA	SFA	SFA developmental delay	SFA intrauterine death risk to mother	SFA/Rape/Social/economic conditions
**Rules for TOP If abortion permissible?**	Even ≥120 days in rape; ≤120 days if life of mother at risk due to SFA. *Egyptian law prohibits abortion under any circums-tance but allowed to save life of mother. Egyptian clergy objected to law legalizing TOP for financial & health reasons [65]	≤120 days	≤120 days only	i. ≤120 days only	i. ≤120 days if foetus will die; SFA incurable	Extremes variations noted;
		≥120 days				TOP ≤120 days if life of mother or health at (18/47 countries); whereas some countries (10/47) allowed TOP on request [50]. 1 in 10 pregnancies resulted in abortion in in the Middle East where TOP is not permitted, unsafe TOP was cause 6% of the maternal mortalities [61]
				ii. SFA/dev delay	ii. ≥120 days if mother certain to die without TOP	
		If proven by 3 medical specialists & consented by both parents	≥120 days if risk to mother’s life proven by 2 medical specialists			
				iii. if it causes extreme suffering to mother and fetus		
					iii. TOP permissible any time if foetus died intra uterine.	
					iv. TOP can only be performed if recommended by 3 physician; if consented by both parents or wife alone if the pregnancy will harm her health or by or any person(s) delegated by parents	
				iv. Mother’s life at risk		Illegal /unsafe TOP rampant in many Middle Eastern countries often requiring hospitalization [62-64]

A recent cross-country study reporting on TOP in Muslim countries from 2013 by Shapiro [50] showed that abortion is acceptable in all countries when there is a threat to the life of the pregnant mother and if 120 days of pregnancy has not lapsed; however, there is considerable heterogeneity in regards to other circumstances such as social or economic situations, the preservation of physical or mental health, foetal anomalies, and rape, in addition to the gestational development of the foetus. Abortion is not allowed in 18 of the 47 countries under any circumstances except for saving the life of the pregnant woman. However, there is considerable diversity between countries, where 10 countries allow TOP *‘on request’*.

The main cause of the problem is the delay in diagnosing the foetal anomaly. Anecdotal evidence suggests that typically affected mothers are usually seen at approximately 18 weeks of pregnancy by general obstetricians due to long waiting lists and crowded facilities. Eighteen weeks is very close (~112 days) to the day of ensoulment, the 120^th^ day of pregnancy. A woman may then be referred to a sub-specialist if she has access to a tertiary healthcare facility. By the time a diagnosis is made, it may be well be past the 140th day of pregnancy. Long waiting lists, crowded facilities and limited numbers of tertiary healthcare facilities, the limited accessibility to a healthcare facility, eligibility issues and similar circumstances may all contribute to a delay and may prevent an early diagnosis. These women will be unfairly required to carry the anomalous foetuses to term; this is a futile and wasteful exercise because their infants will die soon after birth or after a few months, and therefore, this situation does not justify the hardship to the mothers in carrying these foetuses to term. The five guiding principles of Islam and the Qur’anic verses stated in the preceding sections suggest that this should not be the case.

The authors’ request is based on the above arguments and the suggestions of past Islamic scholars that state that foetuses that are incompatible with life at birth are a wasteful and futile effort of the mothers [51-57]. This assumption has formed the basis for a termination prior to 120 days of pregnancy. The same assumption and arguments detailed in the preceding sections are valid points for considering a termination beyond 120 days. The present medical technology can enable the accurate prediction of an anomaly in utero that is incompatible with life. A termination can be performed with a high assurance of accuracy. Therefore, it follows that there is no need for the mothers to suffer in bearing a foetus that results in loss, futility and a waste of effort and resources including physical trauma and psychological and emotional turmoil in compliance with the five guiding principles of Islam and verses from the Glorious Qur’an. Other reasons that support a termination post ensoulment are detailed below.

Second, the reasons for which scholars in the past have ruled against terminating a pregnancy due to foetal anomalies was because of the uncertainty in diagnosing the presence of foetal anomalies. However, recent advances in medicine have now provided investigational modalities with which foetal anomalies can be detected with a very high degree of accuracy [43,55].

Third, in spite of severe foetal anomalies, some foetuses will survive after birth for days or months. The difficult lives these children may experience will cause much pain and suffering for both the affected infants, the infants’ mothers and the immediate members of their families. In addition, there are high costs associated with these births, including the use of scarce health resources such as hospital beds and care that may be better utilised on infants and children with better chances of survival or normal lives. The proper utilisation of scarce resources forms the basis for one of the five guiding principles of Islam that was alluded to in a preceding section [56,57].

Fourth, some religious scholars argue that anomalous foetuses will serve to remind those born normal of the need to be grateful to God, but in reality, a majority of these foetuses will die prematurely either before or soon after delivery; therefore, this argument does not carry water but it causes much hardship and pain to the mother [55,58].

Fifth, religious scholars may maintain that humans were created to worship God, and therefore, all life forms must be allowed their full lease of life. However, as stated in the preceding pgraph, the affected infants may die soon after birth and cannot be anticipated to worship God; in fact, their birth may cause considerable pain and suffering to the mother, so this argument may be unjustified.

Sixth, a guilty consciousness due to religious convictions in the affected mothers may prompt them to carry the severely anomalous foetuses to term unless religious edicts provide them the means to effectively address the situation at the earliest possible time. Most often this occurs due to a misplaced adherence to a self-imposed assumption that TOP is not permissible. More importantly, severe foetal anomalies could lead to pregnancy complications leading to maternal morbidity or worse, mortality. Other complications include psychological trauma, pain and medical problems including a Caesarean section, which may be completely unnecessary considering that the affected infant may not survive [59]. To accentuate this point, the authors cite an instance of a Caesarean section performed on a normal woman who carried an anomalous foetus to term, only to die subsequently of surgical complications. This was an unnecessary death that occurred for a hopeless and futile cause.

Due to the sensitivity of abortion from a religious point of view, mistakes are not permissible in the diagnosis of foetal anomalies. It is documented that the diagnosis of foetal anomalies is usually accurate in tertiary healthcare centres [60]. The Islamic rule pertaining to abortion is strict, and therefore, the termination of a pregnancy based on the current edict of Saudi Arabia as well as that of other countries has to be undertaken after it has been confirmed with complete certainty that a foetal anomaly exists. Anecdotal evidence has shown that mistakes do occur in the diagnosis of foetal anomalies, especially when a single individual is involved in the decision-making; therefore, the opinion of more than one practitioner must be sought, preferably of different but related sub-specialties.

The lack of the permissibility of abortion may drive some to seek an unsafe abortion. Indeed, one in ten pregnancies ends in abortion in the Middle East and North African (MENA) [61] region, which stretches from Morocco to Iran. Of these abortions, at least 6% of the maternal deaths may be attributed to unsafe abortions [62]. In Egypt, one in five obstetric admissions in 1998 were for post-abortion treatment [63], while in Iran over 1,000 unsafe abortions are estimated to take place daily [64]. A WHO [65] report indicates unsafe abortions lead to 47,000 maternal deaths. Indeed when abortion was legalized maternal morbidity and mortality in South Africa was dramatically reduced [66]. The reverse effect was seen when abortion was banned in Romania [67]. The above statistics and the evidence presented underscore the need for Islamic nations to re-visit their policies on abortion.

The available evidence indicates a large disparity in Islamic thinking on TOP, suggesting the need for Muslim nations to collectively resolve the issue of abortion in Islam for a common stand on this matter for the betterment of the Islamic nation at large and for preserving the well-being of women.

Islamic Law, in spite of its more liberal stance, prohibits the termination of a pregnancy solely on the basis of severe foetal anomalies 120 after post fertilisation if the mothers’ life is not at risk. The issue of the termination of a pregnancy past 120 days of pregnancy due to severe foetal anomalies in Islam lies somewhere between the *permissible* and *forbidden*. In other words, it is a “grey area” in the interpretation of the Islamic literature that very few scholars venture into or will be led to discuss. Due to the enormous amount of pain and suffering that the mothers of affected foetuses endure, the authors implore scholars to re-visit and debate the issue further for pregnancies beyond the 120^th^ day. The authors urge Muslim law makers to deliberate and consider abortion past the 120^th^ day of fertilisation to avoid substantial hardship to mothers and family members if it is certain that the severely anomalous foetus will decease after birth or will be severely malformed and physically and mentally incapacitated after birth. If no religious intervention is instituted, the guilt-conscious Muslim society, and in particular the affected mother, may have to endure pain and suffering for years if the anomalous foetuses are left to proceed to term in spite of the knowledge bestowed upon the learned community that has the means at its disposal to prevent such catastrophes. There is at least one reference in the Glorious Qur’an (18:74,80) indicating that termination can be used in extreme circumstances to protect the parents so that they do not have to suffer because of their children (2:233). The reference to the previous two verses (18:74,80) may not find favour with clerics, and their relevance to the current discourse remains to be acknowledged by the relevant authorities. The authors highlight this verse to stimulate a debate on TOP post the 120^th^ day of pregnancy.

The recommendation of the authors is in keeping with the Glorious Qur’anic verses cited in the preceding and subsequent sections, which indicate that a person shall not be required to undergo harm or difficulty due to religion, in particular mothers [Glorious Qur’an, Al- Baqara 2:286]. There are verses in the Glorious Qur’an that remove guilt if a certain course of action is pursued when there is no recourse:

“But if compelled by necessity, neither desiring it nor transgressing bounds,

*there is no sin. Indeed, God is ever forgiving and merciful” [Glorious Qur’an,* Al- Baqara *2:173]*.

“He has chosen you and not placed upon you a religion that causes you difficulty” [Glorious Qur’an, Al-Hajj 22:78].

Those responsible for matters pertaining to the provision of fair and equitable medical care or edicts must ensure that the under-represented mothers’ well-being is protected through ethical medico-legal and religious edicts or guidelines. More importantly, it is crucial for policy-makers and clerics that advise governments on the issue of edicts pertaining to the TOP debate and to formulate effective policies or edicts so that sufferings can be avoided. It is argued the physical well-being takes precedence over religious well-being. Intervention is lawful if it is useful and beneficial, and the performance of a specific medical procedure that benefits the physical well-being of an individual outweighs generalized religious prohibitions [7,68-70]. The authors wish to caution that total perfection cannot be anticipated from edicts and protocol because fallacies do occur and must be anticipated. It cannot be assumed that edicts are a pgon of proper conduct. In light of the existing religious edicts from various countries, the termination of pregnancies on less firm grounds and for less medically sound reasons has to be avoided. Decisions to perform TOP need be undertaken by an institutional committee formed by the institution. The activities of the institutional committee may be overseen by a national committee to ensure that the issues pertaining to abortion are performed in a way that effectively avoids mistakes. This will ensure that abortions take place only in instances permitted not only by religious edicts but also with sound medical judgment and that the decision for the same should be made through the concurrence of members of diverse but related medical fields such as obstetricians, perinatologists, neonatologists, geneticists, and other relevant medical and surgical sub-specialties, including religious and ethical representatives. The job of these two committees will be to oversee the decisions regarding the TOP due to severe foetal anomalies with known poor prognoses. The national committee shall report to the highest health authority in the country. It is also the responsibility of these two committees to maintain a register of pregnancies that were terminated. It should also maintain a record of false diagnoses and complete statistics of the procedures performed for future reference and use. No termination of a pregnancy should take place without permission from these committees. If a medical team and a structured program are developed to address these issues, it will become possible to avoid mistakes and prevent the loss of life because of the abortion of normal foetuses due to errors in judgment.

Contrary to the dictum that Islam has a solution for all ills, policy and law makers in Islamic nations are still grappling with the issue of TOP, which remains largely unresolved. The authors state that this issue has not been resolved because the available decrees on TOP continue to cause affected women considerable pain, stress, and real suffering. Policy and law-makers have to confront this issue to reach a decisive solution to the problem. Women should have an avenue to make an informed decision pertaining to TOP that is guilt-free. Women have to be empowered to effectively address severe incurable and fatal foetal anomalies. It is recognized complexities [70] confronting the issue of TOP are enormous but these have to be resolved as parents and in particular mothers have suffered and continue to suffer. This has gone long enough. It must be viewed as a matter of grave magnitude that needs urgent attention.

Medical practitioners calculate pregnancy from the first day of the last menses. Fertilisation of an ovum takes place in the Fallopian tube, and then, it takes approximately 7 days for implantation in the uterus. Thus, the implantation of the fertilised ovum takes approximately two to three weeks from the day of the last menses, which is followed by ensoulment of the foetus 120 days after fertilisation while some religious school of thought hold that this occurs at 42 days and an embryo is not an individual until passage of 14 days post fertilization [19]. Healthcare providers must calculate the day of ensoulment carefully to avoid unacceptable mistakes. The 120 days mentioned in the hadith texts and the juristic discussions begin from the time of fertilisation, which is equivalent to 134 days (19 weeks) from the last menstrual period. On this basis it is critical practitioners recognize ensoulment begins at 134 days from the last menstrual period and take all necessary measures to detect foetal anomalies early enough to enable parents avail of TOP prior to ensoulment. All major lethal anomalies take place before ensoulment where the initial screening for foetal anomalies is usually performed. These facts are important for healthcare providers if a decision regarding TOP is required. A discussion of the prevalence and prevention of genetic disorders, and a list of genetical abnormalities affecting pregnancies in a consanguineous Muslim society has been described [7,71].

Policy makers are almost always males who may not adequately empathise with women’s health and emotional issues. In matters pertaining to the health and emotional well-being of women, it may be pertinent to engage qualified female Islamic clerics to deliberate meaningfully on such issues alongside their male counterparts in equitable, impartial and rational terms so that decrees and recommendations on crucial issues such as TOP can be formulated to ensure the well-being of women.

Some service providers may harbour reservations against TOP. The 2006 FIGO guidelines [72] on ‘*Conscientious Objection*’ states that a service ‘*provider can decline a service and not be compelled by another health professional to act contrary to their moral conviction or religious belief, except as required by law’* and as delineated in FIGO’s position on conscientious objection. The FIGO guideline further states that ‘*service providers can provide public notices of services they decline to perform on grounds of conscience’*. They may ‘*refer patients that need such services on medical grounds to other providers that do not object to such services provided they are lawful’*. The guideline recognizes the ‘*patient’s right to timely access to medical services when referral to other practitioners is not possible and delay would jeopardize their health and well-being; and in emergency situations provide care regardless of the service provider’s personal objections*’.

## Summary

The single most critical factor in preventing harm to mothers of anomalous pregnancies is time. Early detection and intervention is crucial. Delays will make it impossible for them to undergo TOP due to prevailing laws on TOP. It is therefore critical front line healthcare providers in obstetrics be vigilant at all times to detect anomalous foetuses at the earliest stages so that termination may be offered prior to ensoulment, which carries less guilt for all parties concerned. The authors urge Muslim law makers to also consider abortion post ensoulment if it is certain that the malformed foetus will decease soon after birth or will be severely malformed and physically and mentally incapacitated after birth to avoid substantial hardship that may continue for years for mothers and family members. The authors recommend that an institutional committee governed and monitored by a national committee make decisions pertaining to abortion to ensure that ethics are preserved and mistakes are prevented. Anomalous foetuses must be detected at the earliest possible time to enable an appropriate medical intervention prior to the 120^th^ day.

## Competing interests

The authors have no conflicts of interest whatsoever with regard to funding, commercial interests, etc.

## Authors’ contributions

AM conceived the idea for the manuscript and wrote the first draft of the paper. JA re-wrote the paper with extensive alterations following extensive literature review. Both authors read and approved the final manuscript.

## About the Authors

Abdulrahman Al-Matary is a Professor and a Consultant Neonatologist at the King Fahad Medical City, Riyadh, Saudi Arabia. He obtained basic training in Medicine (MBBS) at King Saud University, Saudi Arabia. He obtained training and specialty in Neonatology at the University of Manitoba, Canada.

Jaffar Ali is a Professor and Consultant Embryologist at the University of Malaya, Kuala Lumpur, Malaysia. He obtained his PhD in Developmental Physiology at the John Curtin School of Medical Research of the Australian National University, Canberra, Australia.

## Pre-publication history

The pre-publication history for this paper can be accessed here:

http://www.biomedcentral.com/1472-6939/15/10/prepub

## Copyright

This manuscript has not been published previously or submitted elsewhere for publication.
